# Supplementary material for: Complexome profiling of the Chlamydomonas psb28 mutant reveals TEF5 as an early PSII assembly factor
Source: Plant Cell. 2025 Mar 17;37(6):koaf055. doi: 10.1093/plcell/koaf055 (PMC12164590; doi:10.1093/plcell/koaf055)
Supplement: koaf055_Supplementary_Data [file koaf055_supplementary_data.zip › psb28_interactive.zip!/plots/intensity/qualityPlots/peptides/PDS1_Cre12.g509650.t1.2.html]

### Cre12.g509650.t1.2

Trivial name: PDS1  
SeqCoverage [median]: 62.40  
Mass: 62.84 kDa  
  
 

| Sequence | MissedCleavage | Mass [Da] | wtIntensity | muIntensity | Score |
| --- | --- | --- | --- | --- | --- |
| ADQSLAK | 0 | 731.38 | 29783.56 | 43152.57 | 188.81 |
| ALAFIDPDR | 0 | 1016.53 | 2913.73 | 8928.66 | 96.25 |
| ALAFIDPDRLSMTVVLTALNR | 1 | 2315.27 | 9093.27 | 33703.43 | 138.75 |
| DIVLNDDGSVK | 0 | 1173.59 | 2921.41 | 6816.58 | 91.87 |
| DYPRPAFETAETFQEAK | 1 | 1998.93 | 0.00 | 6849.56 | 76.16 |
| EHSMIFAMPDSPGEFSR | 0 | 1936.84 | 22789.46 | 55660.21 | 148.70 |
| ELNIEER | 0 | 901.45 | 38800.01 | 108556.18 | 155.82 |
| EYYDTEK | 0 | 946.39 | 0.00 | 732.86 | 94.66 |
| IQFAIGLLPAIIFGQK | 0 | 1728.03 | 0.00 | 1531.14 | 91.03 |
| KLTTVDHLLFSR | 1 | 1428.81 | 17496.68 | 61692.96 | 134.98 |
| LAAEQIVNDYNYK | 0 | 1539.76 | 69595.93 | 202585.09 | 310.69 |
| LAAEQIVNDYNYKGVAPPAR | 1 | 2188.13 | 3622.15 | 3810.76 | 156.11 |
| LFPTEIK | 0 | 846.49 | 7747.34 | 58234.75 | 100.07 |
| LFPTEIKADQSLAK | 1 | 1559.86 | 19946.49 | 62481.76 | 102.52 |
| LLVPDQWKPNPYFSQLK | 1 | 2072.11 | 6805.32 | 0.00 | 84.84 |
| LQWKEHSMIFAMPDSPGEFSR | 1 | 2492.16 | 1718.60 | 0.00 | 58.49 |
| LSMTVVLTALNR | 0 | 1316.75 | 1078.62 | 7044.04 | 140.49 |
| LTTGEVVEGDLYMSAMPVDILK | 0 | 2380.19 | 12518.22 | 0.00 | 119.96 |
| LTTVDHLLFSR | 0 | 1300.71 | 21310.40 | 61403.15 | 131.37 |
| MAFLDGAPPER | 0 | 1202.58 | 9909.41 | 13892.68 | 185.81 |
| NFFLAGDFTK | 0 | 1158.57 | 14383.70 | 22760.02 | 128.28 |
| NNQMLTWPEK | 0 | 1259.60 | 581.87 | 1401.58 | 90.63 |
| PAFETAETFQEAK | 0 | 1467.69 | 1391.63 | 5550.83 | 162.68 |
| PAKPLKVVIAGAGLAGLSAAK | 2 | 1931.19 | 1297.08 | 0.00 | 56.05 |
| QKYLASMEGAIFSGK | 1 | 1628.82 | 5193.64 | 5303.77 | 95.19 |
| QQGVPDRVNEEVFIAMAK | 1 | 2030.03 | 20032.63 | 49050.60 | 91.93 |
| SDEDIIAATMTELER | 0 | 1692.79 | 27068.26 | 73667.26 | 184.03 |
| SMLELVFAPAK | 0 | 1204.65 | 6440.71 | 8977.27 | 159.37 |
| SMLELVFAPAKDWIGR | 1 | 1831.97 | 2323.89 | 0.00 | 47.55 |
| TPIKNFFLAGDFTK | 1 | 1597.85 | 16257.09 | 0.00 | 114.21 |
| TPLSVYESR | 0 | 1050.53 | 71726.16 | 128772.02 | 209.84 |
| VIKTPLSVYESR | 1 | 1390.78 | 3121.02 | 3506.15 | 111.79 |
| VKDIVLNDDGSVK | 1 | 1400.75 | 42712.19 | 78362.01 | 133.48 |
| VKDIVLNDDGSVKHYK | 2 | 1828.97 | 19776.22 | 21186.40 | 115.80 |
| VNEEVFIAMAK | 0 | 1249.64 | 3574.34 | 15476.39 | 192.26 |
| VVIAGAGLAGLSAAK | 0 | 1296.78 | 25767.96 | 75439.13 | 191.53 |
| YLASMEGAIFSGK | 0 | 1372.67 | 32542.13 | 108344.58 | 199.03 |
| YLSDAGHHPIVLEGR | 0 | 1662.85 | 29541.86 | 65178.31 | 115.06 |
| YVEEQDHLSVTQWMR | 0 | 1919.88 | 9482.35 | 32607.86 | 169.40 |
|  |  |  | 611265.4 | 1432660.6 |  |
